# Histopathological and immunohistochemical characterization of lesions in the golden Syrian hamster model of Nipah virus infection (Bangladesh strain)

**DOI:** 10.3389/fvets.2025.1708412

**Published:** 2026-01-19

**Authors:** Kirsty Swan, Francisco Javier Salguero, Alison Bird, Laura Hunter, Chelsea Kennard, Stephen Findlay-Wilson, Emma Kennedy, Sarah Kempster, Neil Almond, Stuart Dowall, Inés Ruedas-Torres

**Affiliations:** 1 United Kingdom Health Security Agency (UKHSA), Salisbury, United Kingdom; 2 Medicines and Healthcare Products Regulatory Agency (MHRA), London, United Kingdom

**Keywords:** Bangladesh strain, golden Syrian hamster, histopathology, immunopathogenesis, Nipah virus

## Abstract

Nipah virus (NiV) is recognised as a priority pathogen with pandemic potential by the World Health Organisation (WHO). The NiV-Bangladesh strain (NiV-B) has been associated with recent outbreaks in different districts of Bangladesh and the state of Kerala (India), and it is suggested to be more pathogenic and lethal than the NiV-Malaysian strain (NiV-M). In this study, we aimed to describe the clinical signs and pathology of NiV-B using the golden Syrian hamster model following intranasal (IN) and intraperitoneal (IP) inoculation with different doses and to compare with prior NiV-M results. For this purpose, we selected samples from NiV-B-infected animals that were submitted for H&E evaluation, immunohistochemistry (IHC), *in situ* hybridisation (ISH) (RNAscope technique), and multiplex immunofluorescence (mIF). The absence of neurological signs was observed in NiV-B-infected animals compared with those that were NiV-M-infected. Except for the brain, which did show only mild lesions, histopathological analysis of NiV-B demonstrated similar pathology and viral RNA in the lung, spleen, and liver compared to those of NiV-M infected animals, with the lung being the main affected organ. Pulmonary lesions consisted of areas of broncho-interstitial pneumonia associated with high cell death activation (caspase-3), proliferation (Ki67), and abundant intralesional macrophages (Iba1) and T cells (CD3). Differential upregulation of the cytokine IL-6 was observed in the lung from NiV-B compared with NiV-M infected animals. Moreover, we demonstrated the wide distribution of the NiV receptor ephrin B2 in endothelial cells, neurons, smooth muscle, epithelial cells, macrophages/type II pneumocytes, and T cells.

## 1 Introduction

Nipah virus (NiV) is a newly emerging negative-sense single-stranded RNA-enveloped zoonotic pathogen (1–3). It belongs to the *Henipavirus* genus, together with Hendra virus and Ghana virus, within the family *Paramyxoviridae* (4). NiV has been recognised as a priority pathogen with pandemic potential and a global health threat by the World Health Organisation (WHO) due to being highly pathogenic and having no licensed prophylactics or treatments currently available for its use in humans, although several vaccines are already in clinical trials (5, 6).

This virus, much like others within the *Paramyxoviridae* family, has fruit bats (*Pteropodidae*) as its natural reservoir (1, 3, 7). *Pteropodidae* are known to be able to travel large distances and are widely distributed across regions of Africa, Australia, and South and Southeast Asia (8, 9). Moreover, NiV has the ability to infect a wide range of host species, including various livestock, which increases the risk for future spillover events and outbreaks (10).

NiV was originally identified in 1998 in Kampung Sungai Nipah, Malaysia, a village where it infected pigs, an intermediate amplifying host, causing the disease to be initially called “porcine respiratory and encephalitic syndrome (PRES)” or “barking pig syndrome (BPS)” (3, 11–13). Many pig farmers who were exposed to infected animals reported clinical signs of respiratory disease and encephalitis (12, 14) with a high mortality rate. Since then, there have been numerous outbreaks throughout Asia (Malaysia, Bangladesh, India, and Singapore) from two genetically distinct strains, the Malaysia strain (NiV-M) and Bangladesh strain (NiV-B) (15, 16). NiV-B is believed to be caused by food-borne transmission primarily as a result of the consumption of contaminated raw date palm sap and fruit (1, 17). The recurrent annual outbreaks in South Asia have occurred seasonally, between the months of December and April, coinciding with date palm sap harvesting season (18, 19).

A closely related virus, Hendra virus (HeV), emerged in Australia primarily in horses 4 years prior (1994) to the first outbreak of Nipah (20, 21). Currently, there is a commercially available and effective vaccine against Hendra virus (Equivac^®^ HeV) for use in horses (22), but no vaccines or antivirals have been developed for use in humans. Thanks to the accelerated R&D for henipaviruses, vaccine development is in progress with several candidates in various stages of clinical testing (23, 24). For NiV, there are currently four vaccine candidates on Phase I clinical trial that incorporate the viral glycoproteins F and G, as the primary NiV antigens (25).

Further research into the understanding of the pathogenesis of Henipaviruses is vital for the development of countermeasures. An appropriate animal model must be utilised to demonstrate a similar NiV disease to that seen in humans. Previously identified animal models for henipaviruses include pigs, horses, mice, ferrets, cats, guinea pigs, golden Syrian hamsters, and non-human primates (African green monkeys and cynomolgus macaques) (26–32). Prior studies based on the golden Syrian hamster and African green monkey models largely discuss the pathology of the NiV-M (33–37); however, there is limited research focused on NiV-B.

NiV primarily infects respiratory epithelial cells, central nervous system cells, and vascular endothelial cells (37, 38). This cell tropism is correlated with the expression of the henipavirus receptors Ephrin B2 and Ephrin B3 (39). Ephrin B2 is expressed in the majority of human tissues to different degrees, the highest expression being in the lung (40), whereas Ephrin B3 is more expressed in different areas of the central nervous system (40, 41). We aim to describe the *in situ* expression of the Ephrin B2 receptor in the hamster model and discuss whether its expression could contribute to the viral pathogenesis.

NiV-B is known to be significantly more pathogenic in humans than NiV-M, with a case fatality rate (CFR) of 70.97% (cases: 335 and deaths: 237) as of 2023 (42) compared to ~40% (cases: 265 and deaths: 105) (33, 37, 43). Moreover, it has been reported that patients infected with NiV-B present more severe respiratory disease, and only a few cases infected with this strain present myoclonus as a clinical manifestation in comparison to NiV-M (44–46). Given the epidemiological and clinical differences in humans, this study aimed to delve into the pathogenesis of NiV-B in the golden Syrian hamster model and compare new data with our previous results focused on the NiV-M model using various histopathological techniques.

## 2 Materials and methods

### 2.1 Animal experiments and sample collection

Archived tissue samples from two different animal experiments were used to perform this study. Animal experiments were carried out at the United Kingdom Health and Security Agency (UKHSA), Porton Down, within an ACDP Containment Level 4 (Biosafety Level 4) laboratory. This study was conducted in compliance with local and institutional legislation and regulations, with a corresponding project license approved by UKHSA’s Animal Welfare and Ethical Review Body (AWERB) and under the authority of a UK Home Office-granted license.

A total of 56 golden Syrian hamsters of >8 weeks of age were challenged with NiV-B via intranasal (IN) or intraperitoneal (IP) routes with differing infection doses. Animals were housed within half-suited rigid isolator cages with *ad libitum* access to food and water. When the scheduled endpoint of the study [2-, 4- and 21-days post-challenge (dpc)] or the humane endpoint (20% of weight loss or neurological signs) was reached, the hamsters were humanely euthanised with an overdose of intraperitoneally administered sodium pentobarbital while under inhaled halothane terminal anaesthesia. The animals were regularly monitored post-infection for daily weight and temperature checks, along with clinical signs checked twice daily, which increased to four times a day following the appearance of any clinical signs. Clinical signs of disease were scored based on the following criteria: 0, healthy; 1, behavioural change, eyes shut; 2, ruffled fur; 3, wasp-waisted, arched back, dehydrated; 5, laboured breathing; 8, ataxia; and 10, immobility, neurological signs, and paralysis. A cumulative score combining all observed signs was then assigned for each animal at each time point.

In total, samples from 15 animals were selected for histopathology and NiV RNAscope *in situ* hybridisation (ISH) analysis, including five different experimental groups (IN, TCID_50_ 10^5^, TCID_50_ 10^4^, and TCID_50_ 10^2^; IP, TCID_50_ 10^3^, and TCID_50_ 10^2^) with three animals from each group. Details of animal experiments are summarized in Table 1. Additionally, three naïve animals, sham-inoculated with phosphate-buffered saline (PBS), were included as a control group. Samples from lung and brain were used for further immunohistochemistry (IHC), IL-6 mRNA RNAscope, and multiplex immunofluorescence (mIF) analysis.

**Table 1:** Details of animal experimentation.

Group (*n* = 3)	Route of infection	Dose (TCID_50_)	DPC (humane endpoint)
1	IN	10^5^	2.5
2	IN	10^4^	2.5
3	IN	10^2^	4, 5, 6.5
4	IP	10^3^	6, 6.5, 9
5	IP	10^2^	6, 6.5, 17

There were three golden Syrian hamsters in each group, making up a total number of 15 selected animals. Route of infection, dose, and dpc. IN, intranasal; IP, intraperitoneal; DPC, days post-challenge.

The NiV [Bangladesh strain: prototype 200401066 was kindly provided by the US Centers for Disease Control and Prevention (CDC)] and the hamsters were supplied by Envigo (Hillcrest, United Kingdom), a UK Home Office-approved supplier.

### 2.2 Histopathology

At *post-mortem* examination, tissue samples were collected from the lung, spleen, liver, nasal turbinates, and brain, and fixed by immersion in 10% neutral-buffered formalin (NBF) for 3 weeks and routinely processed in paraffin wax [nasal turbinate samples were decalcified in OSTEOSOFT (Sigma-Aldrich, Missouri, United States) prior to processing]. Samples were sectioned at 4 μm and stained using haematoxylin and eosin (H&E). The stained slides were then digitally scanned by a Hamamatsu S360 digital slide scanner (Hamamatsu Photonics K.K., Shizuoka, Japan) and evaluated subjectively by a pathologist using the NDP.view2 software (Hamamatsu Photonics K.K., v2.8.24). The pathologist was blinded to treatment group details, and the slides were randomised prior to examination to prevent bias (blind evaluation). Subsequent analysis and evaluation involved the semi-quantitative histopathological score of the severity of lesions present in the lung, brain, spleen, liver, and nasal turbinates (0 = within normal limits; 1 = minimal histopathological lesions; 2 = mild histopathological lesions; 3 = moderate histopathological lesions and 4 = marked/severe histopathological lesions), as previously described by our group (27, 36, 47). In the lung, the severity of the broncho-interstitial pneumonia was evaluated and recorded. In the brain, the presence of meningitis and perivascular cuffing was scored. In the liver, the presence of inflammatory infiltrates was also evaluated. In the spleen, the presence of polymorphonuclear cell infiltrates and the level of lymphoid depletion were recorded. Finally, in the nasal turbinates, the presence of inflammatory infiltrates, nasal exudate, and necrosis of the epithelia was also semi-quantitatively scored and recorded. In the brain, spleen, and nasal turbinates, the sum of all scored parameters for these organs was considered the cumulative score.

### 2.3 NiV RNA and IL-6 mRNA *in situ* hybridisation (RNAscope)

*In-situ* hybridisation (ISH) RNAscope was performed on all samples to detect and evaluate the distribution of the NiV RNA. The technique was executed automatically on the Leica BOND-RX (Leica Microsystems, Milton Keynes, United Kingdom). The slides underwent pre-treatment using hydrogen peroxide for 10 min, heat-induced target retrieval for 15 min at 95 °C with epitope retrieval 2 solution (Leica Microsystems), and a protease step for 15 min (42 °C) (Advanced Cell Diagnostics, CA, United States). The samples were then incubated for 2 h at 42 °C following application of the NiV-specific probe (Cat No. 439258, Advanced Cell Diagnostics). Adhering to the manufacturer’s instructions, the signal was amplified using RNAscope 2.5 LS Detection Kit-RED (Catalogue Number: 322150. Advanced Cell Diagnostics), and the chromogen was applied using the Leica Bond Polymer Refine Red Detection Kit (Catalogue Number: DS9390. Leica Microsystems). Similarly, the manual RNAscope technique was used to label interleukin-6 (IL-6) mRNA (Cat No. 1062321-C1) in all lung samples. Briefly, the slides underwent pretreatment using hydrogen peroxide for 10 min (RT), target retrieval for 15 min (98–101 °C), and protease plus for 30 min (40 °C) (Advanced Cell Diagnostics) before the probe was incubated for 2 h (40 °C), and the signal was amplified using RNAscope 2.5. HD Reagent kit—RED (Catalogue Number: 322350. Advanced Cell Diagnostics). Each ISH run included adequate positive and negative controls.

All RNAscope-stained slides were mounted using EcoMount (Biocare Medical, CA, United States) and digitally scanned with the Hamamatsu S360 digital slide scanner (Hamamatsu Photonics K.K.). The slides were subjected to image analysis using Nikon NIS-Ar software (Nikon, Instruments Inc., NY, United States) to calculate the percentage of positively stained area for viral RNA and IL-6 mRNA present in each tissue section.

### 2.4 Immunohistochemistry

In order to study NiV pathogenesis, immunohistochemistry (IHC) against the main NiV receptor (Ephrin-B2) was performed in selected samples with high levels of viral RNA and naïve animals. Additionally, lung and brain samples were stained against CD3 (T cells), Iba1 (macrophages/microglia), and GFAP (astrocytes) to identify the cellular components of lesions and inflammatory infiltrates. Furthermore, samples were stained to evaluate cell proliferation (Ki67) and apoptosis (cleaved caspase-3). The IHC technique was performed on the Leica BOND-RX (Leica Microsystems), and the methodology is described in Table 2. Antigen retrieval was either via BOND epitope retrieval solution 1 (ER1, pH 6.0) for 20 min at 95 °C or epitope retrieval solution 2 (ER2, pH 9.0) for 20 min at 95 °C (Table 2). Followed by blocking using superblock (TBS) blocking buffer (Thermo Scientific, United States), the antibodies were then incubated for 30 min. BOND polymer refine detection kit (Leica Microsystems) was applied for immunostaining, and the slides were dehydrated and mounted using Dako mounting medium (Agilent, Santa Clara, United States). Except for the Ephrin B2, all slides were submitted to quantification to calculate the percentage area of positive staining by using Nikon NIS-Ar software (Nikon). For Ephrin B2, due to the ubiquitous staining, the digital quantification of this marker was not performed; a subjective evaluation was done instead.

**Table 2:** Summary of immunohistochemical (IHC) methods: primary antibody, dilution, blocking solution, antigen retrieval, and source.

Primary antibody (clone)	Type of antibody	Dilution	Blocking solution	HIER	Source
CD3	Polyclonal	1:50	Superblock^a^	ER1 20 min^b^	Agilent Dako, CA, United States
GFAP (GA5)	Monoclonal	1:300	Superblock^a^	ER1 20 min^b^	FluidIGM, CA, United States
Iba1	Polyclonal	1:750	Superblock^a^	ER1 20 min^b^	FujiFilm Wako, Neuss, Germany
Ki67	Polyclonal	1:300	Superblock^a^	ER1 20 min^b^	abCAM, Cambridge, United Kingdom
Caspase-3 (ASP175) (D3E9)	Monoclonal	1:100	Superblock^a^	ER1 20 min^b^	Cell Signaling, MA, United States
Ephrin B2 (JM53-21)	Monoclonal	1:200	Superblock^a^	ER2 20 min^c^	Invitrogen, MA, United States

HIER, heat-induced epitope retrieval.

a Superblock (TBS) blocking buffer (Thermo Scientific, United States).

b BOND epitope retrieval solution 1 (pH 6.0).

c BOND epitope retrieval solution 2 (pH 9.0).

### 2.5 Multiplex immunofluorescence

To investigate the host–pathogen interactions of NiV within the lung and brain, a multiplex immunofluorescence (mIF) technique was used. Selected samples from the brain and lung, which showed a high presence of NiV RNA and severe lesions (group 5), were utilised to perform mIF. The mIF technique employed some of the primary antibodies used in the IHC technique (Table 3). The Opal 6-Plex Detection Kit (Catalogue Number: NEL821001KT, Akoya Biosciences, MA, United States) was used for five-plex staining using the Leica BOND-RX (Leica Microsystems). Details of the Opal fluorochrome tag applied to each primary antibody are described in Table 3. One autofluorescence unstained control slide was included in the experiment to isolate the autofluorescence spectrum.

**Table 3:** Summary of multiplex immunofluorescence (mIF) techniques: primary antibody and opal tag detail.

Primary antibody	Primary antibody dilution	Source	HIER	Opal	Opal dilution
Anti-NiV nucleoprotein	1:200	Invitrogen, MA, United States	ER1 20 min^a^	Opal 520 (green)	1:150
CD3	1:50	Agilent Dako, CA, United States	ER1 20 min^a^	Opal 570 (yellow)	1:150
Ephrin B2	1:200	Invitrogen, MA, United States	ER2 20min^b^	Opal 620 (orange)	1:150
GFAP	1:300	FluidIGM, CA, United States	ER1 20 min^a^	Opal 620 (orange)	1:150
Iba1	1:200	FujiFilm Wako, Neuss, Germany	ER1 20 min^a^	Opal 690 (red)	1:150
Dapi	1:10	—	ER2 20 min^b^	—	—

HIER, heat-induced epitope retrieval.

a BOND epitope retrieval solution 1 (pH 6.0).

b BOND epitope retrieval solution 2 (pH 9.0).

Following staining, the slides were digitally scanned with the multispectral camera of a PhenoImager^®^ HT (Akoya Biosciences) and visualised with PhenochartTM software (Akoya Biosciences). The slides were later analysed using inForm^®^ analysis software (Akoya Biosciences) to identify the cell population and co-location. For this, machine learning classifiers (tissue segmentation, cell segmentation, and cell phenotyping) were trained and applied to the images. The methodology applied to perform the image analysis in the lung and brain with the inForm software is shown in Supplementary Figures 1, 2. A total of 15 regions of interest (ROI) were selected and batch-analysed. The data were further consolidated and summarized using phenoptrReport (Akoya Biosciences) in RStudio (R version 4.2.2, MA, United States) to create reports with the cell counting data and co-location in each selected field.

All histopathology, ISH, IHC, and mIF studies were conducted in an ISO9001:2015 compliant laboratory.

Animals were required to be humanely euthanized at different time points based on clinical endpoints, resulting in markedly different durations of infection. Consequently, the groups are not directly comparable with respect to disease progression, and statistical analysis was not included in this study.

## 3 Results

### 3.1 Clinical observations

**Figure 1:**
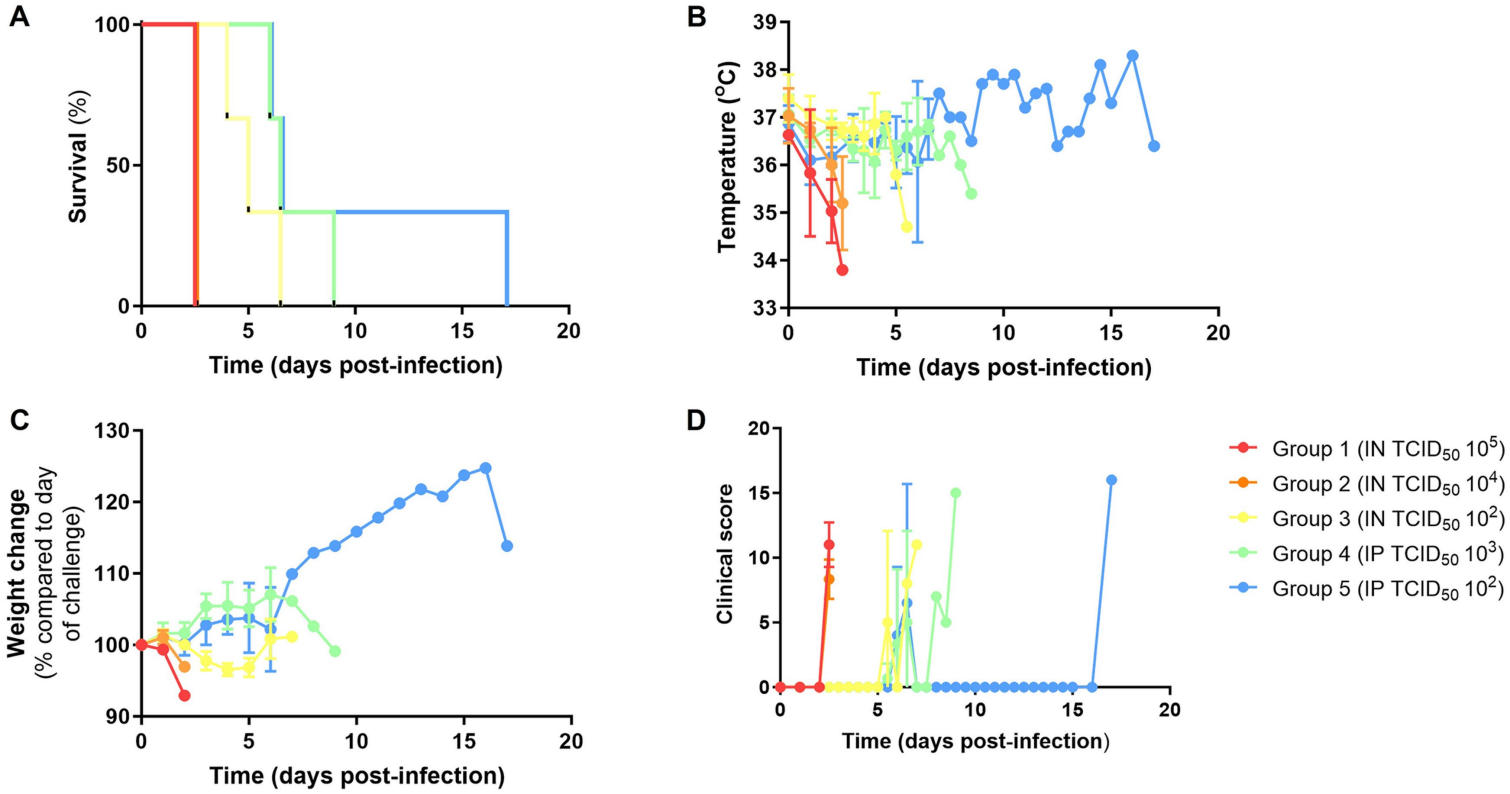
Clinical parameters from hamsters challenged intranasally (IN) and intraperitoneally (IP) with NiV-B at different doses. **(A)** Kaplan–Meier survival plot. **(B)** Temperature (°C). **(C)** Weight change compared to the day of the challenge. **(D)** Clinical score. Data shows mean values with error bars denoting standard error from *N* = 3 (TCID_50_ 10^5^, TCID_50_ 10^4^, and TCID_50_ 10^2^; IN route) and *N* = 3 (TCID_50_ 10^3^ and TCID_50_ 10^2^; IP route).

All NiV-B infected animals met humane clinical endpoints according to the severity of the disease (Table 1), with infected animals meeting the humane endpoint between day 2 and 9 post-infection, except for one animal IP-challenged with the lowest dose (10^2^), which survived until day 17 of the study (Figure 1A). Severe hypothermia and weight loss were observed in IN-inoculated animals, whereas IP-inoculated animals showed more stable temperatures and weight gain (Figures 1B,C).

Lethargy and severe respiratory signs (dyspnoea and tachypnoea) were observed in most of the infected animals. No neurological signs were observed in any of the animals, except for one animal from group 4 (IP, 10^3^), which experienced tremor. Clinical signs were observed from day 2 post-infection in animals inoculated IN high dose (10^5^ and 10^4^) and from day 5 in those infected IN low (10^2^) dose and IP (Figure 1D).

### 3.2 Histopathological lesions

**Figure 2:**
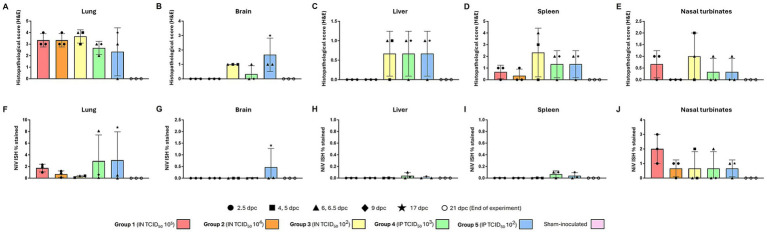
Quantitative results of histopathological scoring and *in situ* hybridization (ISH) in various organs of animals from different experimental groups. Histopathological score in **(A)** lung, **(B)** brain, **(C)** liver, **(D)** spleen, and **(E)** nasal turbinates. Percentage area positively stained following ISH in **(F)** lung, **(G)** brain, **(H)** liver, **(I)** spleen, and **(J)** nasal turbinates. Column and whisker plots demonstrate the median and range with data points indicating individual animals. *N* = 3 animals per experimental group.

**Figure 3:**
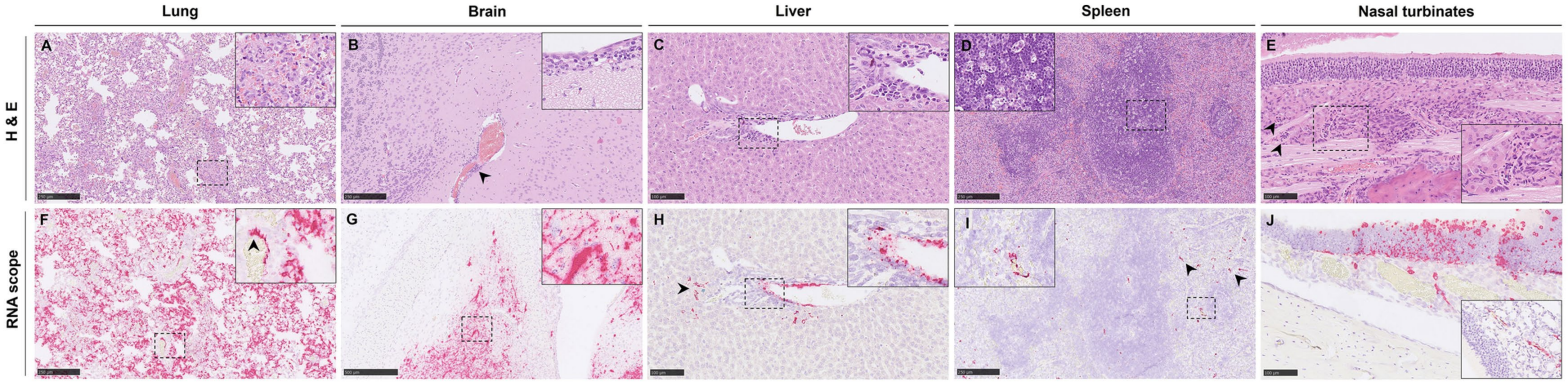
Representative histopathological lesions (H&E) and NiV ISH RNAscope in tissues from NiV-B infected animals. **(A)** Lung section from a group 5 animal showing moderate interstitial pneumonia characterized by thickening of the alveolar walls. **(B)** Brain section from a group 5 animal showing perivascular cuffing (arrowhead) and mild meningitis (inset). **(C)** Liver section from a group 5 animal showing inflammatory infiltrates at the periportal level. The inset shows higher magnification. **(D)** Spleen from a group 4 animal showing tingible body macrophages (cellular death). The inset shows higher magnification. **(E)** Nasal turbinates from a group 3 animal showing mild inflammatory infiltration (arrowheads). The inset shows higher magnification. **(F)** Same RNAscope as **(A)**, showing intense positivity in areas of interstitial pneumonia and endothelium. The inset shows viral RNA in inflammatory cells and endothelial cells (arrowhead). **(G)** Same RNAscope as **(B)**, showing intense positivity in the midbrain. The inset shows viral RNA within neurons and neurophils. **(H)** Same RNAscope as **(C)** showing viral RNA within the inflammatory infiltrates (arrowhead) and endothelial cells. The inset shows a higher magnification of positive endothelial cells. **(I)** Same RNAscope as **(D)** showing viral RNA in immune cells from the red (arrowheads) and white pulp. The inset shows a higher magnification of positive immune cells. **(J)** Same RNAscope as **(E)** showing viral RNA in epithelial and sustentacular cells within the olfactory mucosa. The inset shows RNAscope from a group 5 animal showing viral RNA in endothelial cells. Scale bars **C,E,H,J** = 100 μm; **A,B,D,F,I** = 250 μm; **G** = 500 μm.

Tissues from sham-inoculated animals did not show any histopathological lesions. In the lung, no differences in the severity of the lesions were found between the different routes and doses of infection (Figure 2A). Lesions consisted of moderate to severe multifocal to coalescing areas of broncho-interstitial pneumonia characterized by thickening of the alveolar walls and type II pneumocyte hyperplasia with necrosis of alveolar and bronchiolar epithelium (Figure 3A). Presence of heterophils, cell debris, alveolar macrophages, and mucus within the alveolar, bronchiolar, and bronchial luminae was observed (Figure 3A, inset).

In the brain, only mild lesions were found in animals inoculated with low doses (Figure 2B), especially in group 5 (IP 10^2^). Lesions consisted of mild meningitis (Figure 3B, inset) accompanied by occasional perivascular cuffing (Figure 3B, arrowhead).

In the liver, lesions were found only in IN-inoculated animals with low dose (group 3) and IP-inoculated animals (Figure 2C). The main observed lesion was the presence of periportal inflammatory infiltrates composed primarily of mononuclear and some polymorphonuclear cells (Figure 3C, inset).

Mild-to-moderate lymphoid depletion and cell death (apoptosis) (Figure 3D, inset) were observed in the spleen from all infected groups (Figure 2D), together with polymorphonuclear infiltrates mainly within the splenic red pulp.

Lesions in the nasal turbinates consisted of minimal diffuse inflammatory infiltration (Figure 3E, arrowheads and inset) composed mainly of mononuclear cells, observed in animals inoculated through both routes (Figure 2E).

### 3.3 NiV RNA distribution in tissue

In the lung, viral RNA was observed in areas of broncho-interstitial pneumonia, particularly in those IN inoculated with a high dose (group 1, 10^5^) and those IP inoculated that survived until the experimental endpoint (17 dpc) (Figures 2F, 3F). Viral RNA was associated with inflammatory cells (Figure 3F, inset) within areas of interstitial pneumonia and endothelial cells of arterioles and venules (Figure 3F, inset, arrowhead).

Viral RNA in the brain was only detected in a few IP-inoculated animals (Figure 2G), showing moderate amounts of viral RNA in one animal inoculated with the 10^2^ dose that survived until the end of the experiment (17 dpc) (Figure 3G). Viral RNA was detected within neurons and neuropil (Figure 2G, inset) within the mid-brain, olfactory bulb, and meninges.

In the liver and the spleen, viral RNA was only detected in IN-inoculated animals with low dose (group 3) and all IP-inoculated animals (Figures 2H,I). In these animals, viral RNA was observed within Kupffer cells, endothelial cells (Figure 3H, inset), and sinusoids in the liver (Figure 3H, arrowhead). In the spleen (Figure 2I), viral RNA was detected in immune cells, mostly within the red pulp (Figure 3I, inset and arrowheads).

Viral RNA was detected in epithelial and sustentacular cells in IN-inoculated animals (Figure 3J) and endothelial cells in IP-inoculated animals (Figure 3J, inset) within the olfactory mucosa from all the inoculated groups (Figure 2J). Nasal turbinates from IN-inoculated animals with a high dose (10^5^) showed higher amounts of viral RNA (Figures 2J, 3J).

### 3.4 Distribution of NiV receptor Ephrin B2

**Figure 4:**
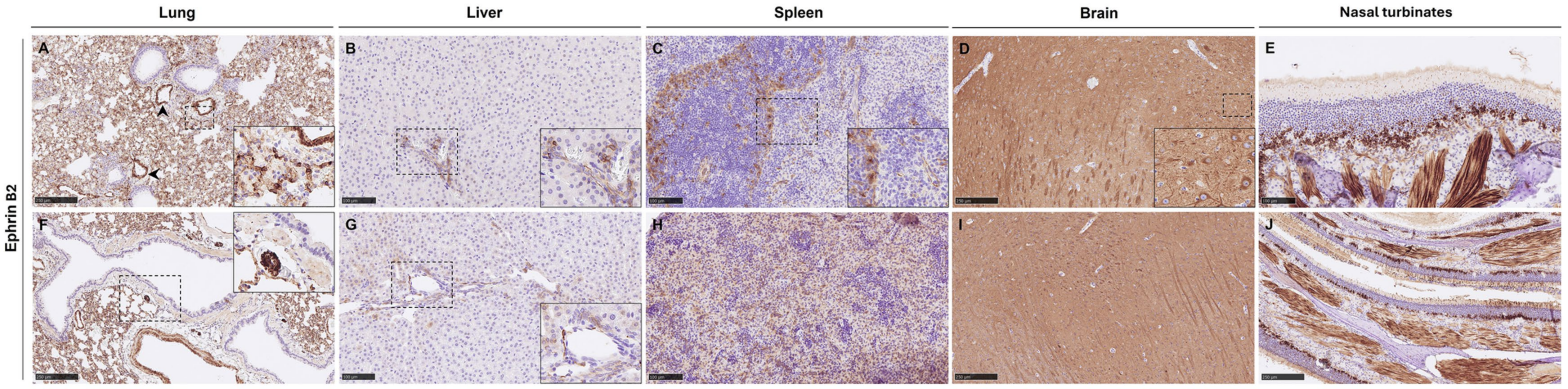
Representative images of immunohistochemistry (IHC) against Ephrin B2 in the different tissues. **(A)** Lung from a group 5 animal showing Ephrin B2 expression in immune cells of the alveolar walls and in endothelial cells and smooth muscle cells from blood vessels (arrowheads). The inset shows higher magnification. **(B)** Liver section from a group 4 animal showing endothelial cells and immune cells expressing Ephrin B2. **(C)** Spleen section from a group 4 animal showing Ephrin B2 staining in immune cells within the white pulp and endothelial cells within the red pulp. The inset shows higher magnification. **(D)** Brain from a group 4 animal showing positive Ephrin B2 staining diffusely distributed in the parenchyma with stronger positivity in neurons and astrocytes. The inset shows a higher magnification of positive neurons. **(E)** Nasal turbinates from a group 5 animal showing Ephrin B2 staining in olfactory cells and connective tissue of the lamina propria. **(F)** Lung from a sham-inoculated animal showing Ephrin B2 expression in immune cells of the alveolar walls, in endothelial cells, smooth muscle cells from blood vessels, and submucosal glands of the bronchi (inset). **(G)** Liver section from a sham-inoculated animal showing endothelial cells and immune cells expressing Ephrin B2. **(H)** Spleen section from a sham-inoculated animal showing Ephrin B2 staining in immune cells within the white pulp and endothelial cells within the red pulp. **(I)** Brain from a sham-inoculated animal showing positive Ephrin B2 staining diffusely distributed in the parenchyma with stronger positivity in neurons and astrocytes. **(J)** Nasal turbinates from a sham-inoculated animal showing Ephrin B2 staining in olfactory cells and connective tissue of the lamina propria. Scale bars **B,C,E,G,H** = 100 μm; **A,D,F,I,J** = 250 μm.

Ephrin B2 showed a widespread distribution in all the tissues, but the lung and brain displayed a more evident expression of the receptor. In the lung, Ephrin B2 was observed in endothelial cells and smooth muscle cells from blood vessels (Figure 4A, arrowheads), immune cells of the alveolar walls (Figure 4A, inset), and submucosal glands of the bronchi (Figure 4F, inset). Stained endothelial cells and immune cells expressing Ephrin B2 were also evident in the liver (Figures 4B,G). In the spleen, Ephrin B2-positive cells were immune cells, especially present in the white pulp and endothelial cells (Figure 4C, inset). In the brain, Ephrin B2 positive staining was observed diffusely distributed in all the parenchyma, but neurons and astrocytes showed a stronger positive staining (Figure 4D, inset and 4I). Finally, in the nasal turbinates, Ephrin B2 positive staining was observed in olfactory cells and the smooth muscle of the lamina propria (Figures 4E,J). No differences were observed between the expression of Ephrin B2 in tissues from infected (Figures 4A–E) and sham-inoculated animals (Figures 4F–J).

### 3.5 Cell phenotyping of lesions within the brain

**Figure 5:**
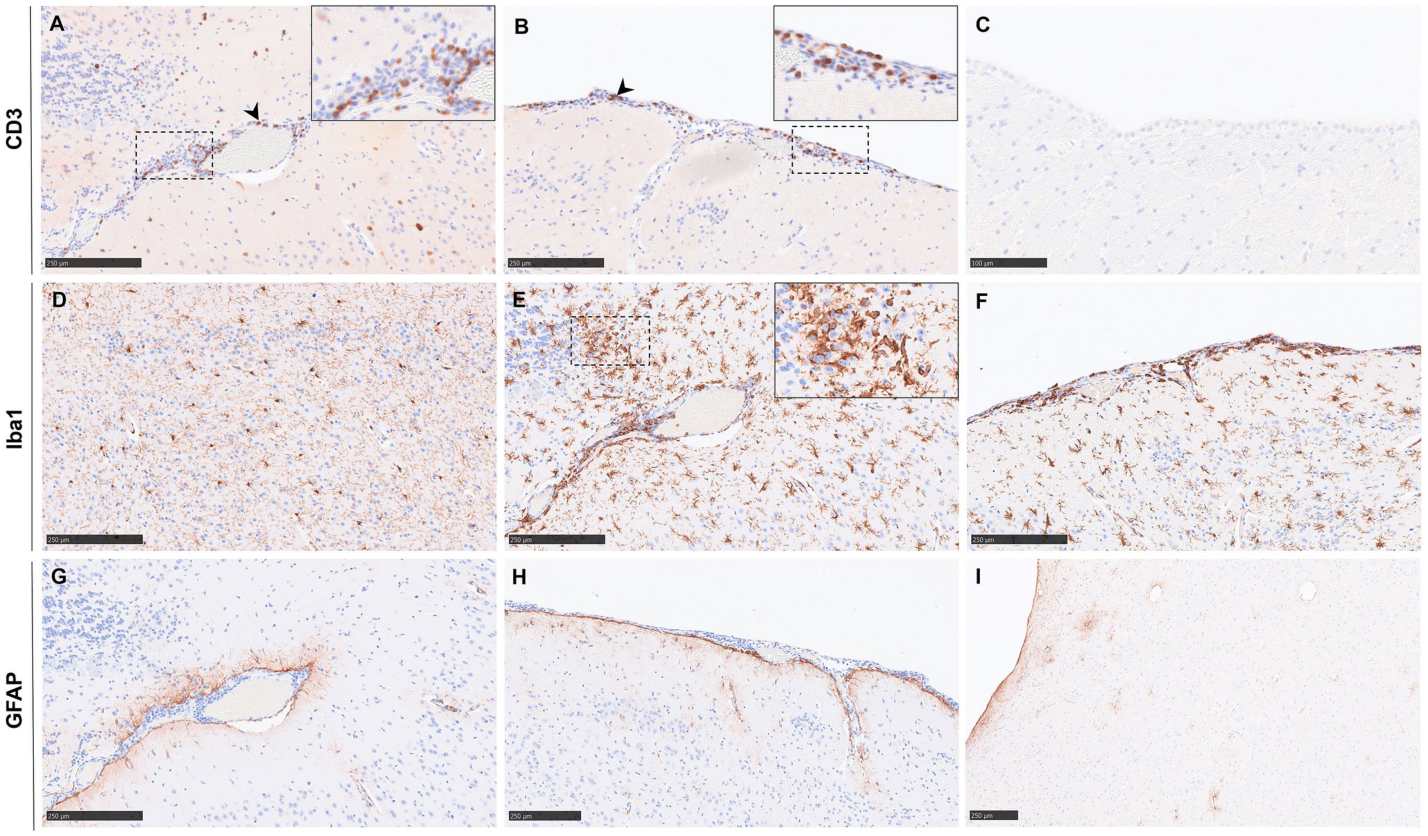
Representative images of immunohistochemistry (IHC) in the brain against CD3, Iba1, and GFAP. **(A)** Brain from a group 5 animal showing CD3^+^ cells found in areas of encephalitis and perivascular cuffing. The inset shows a higher magnification. **(B)** Meninges from the same animal as **(A)** showing positive staining against CD3. The inset shows a higher magnification of CD3^+^ cells. **(C)** Brain of a sham-inoculated animal stained against CD3 showing no positive staining. **(D)** Brain from a group 3 animal showing Iba1^+^ cells diffusely distributed through the midbrain. **(E)** Brain from an animal from group 5 showing microglia^+^ cells. The inset shows a higher magnification of microglia^+^ cells. Arrowhead shows perivascular cuffing. **(F)** Meninges from the same animal as **(E)** showing positive staining against Iba1. **(G)** Brain from a group 5 animal showing astrocytes^+^ cells surrounding the perivascular cuffing. **(H)** Meninges from the same animal as **(G)** showing GFAP^+^ cells. **(I)** Brain of a sham-inoculated group animal stained against GFAP. Scale bars **C** = 100 μm; **A,B,D–I** = 250 μm.

Except for one sample from an animal from group 5 (IP 10^2^) that survived until the end of the experiment (17 dpc) and showed the most severe histopathological lesions and high viral RNA, CD3 staining (T cells) was practically absent in all the brain samples. Hence, CD3-positive staining was not quantified within the brain. In that aforementioned animal, CD3-positive cells were detected in the perivascular cuffs (Figure 5A, inset), meningitis foci (Figure 5B, arrowhead), and areas of encephalitis (Figure 5A). Sham-inoculated animals showed no CD3-positive staining in the brain (Figure 5C).

**Figure 6:**
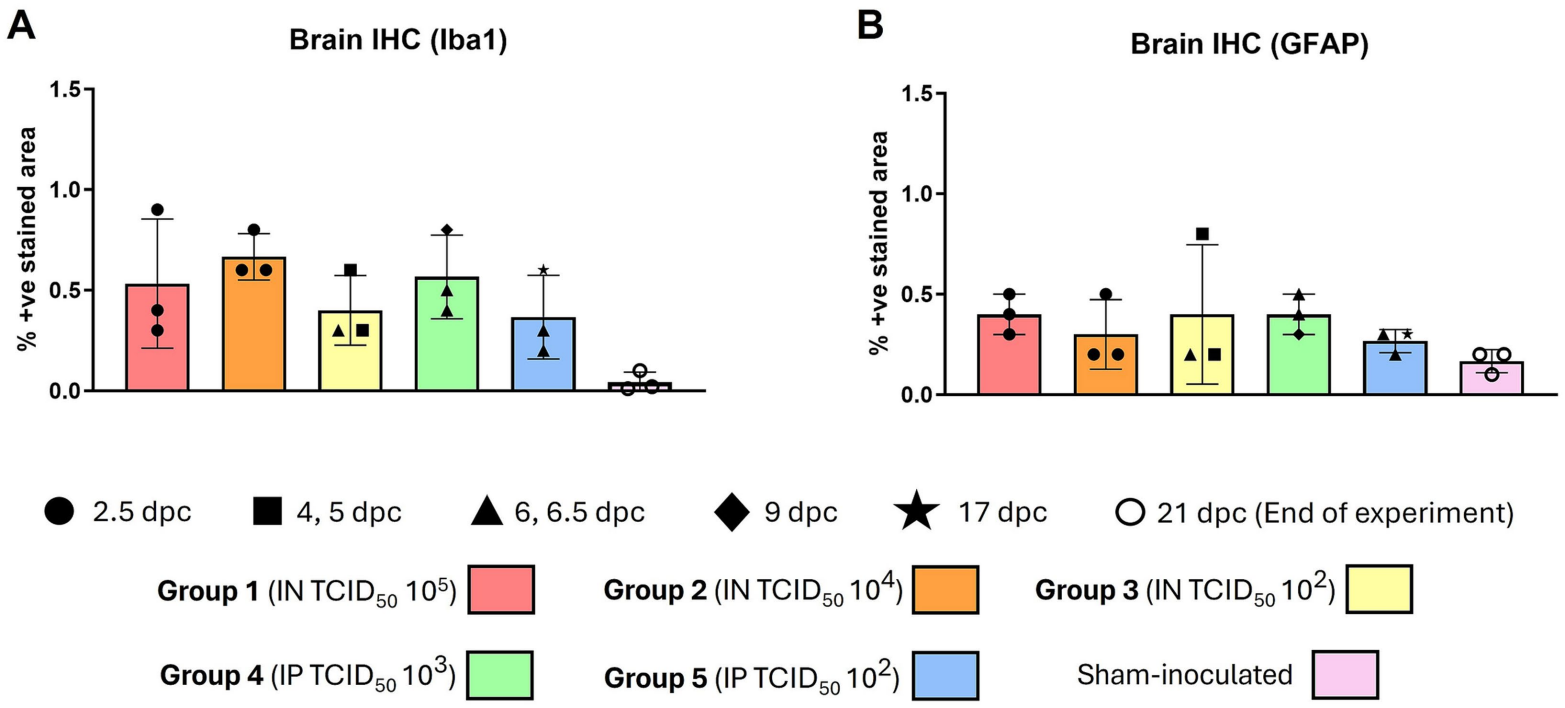
Quantitative results of Iba1 and GFAP immunohistochemistry (IHC) in the brain of animals from different experimental groups following image analysis. **(A)** Graph representing the quantification of Iba1 in the brain. **(B)** Graph representing the quantification of GFAP in the brain. Column and whisker plots demonstrate the median and range with data points indicating individual animals. *N* = 3 animals per experimental group.

IHC quantification results of cell populations (Iba1 and GFAP positive staining) recorded within the brain are displayed in Figure 6. Iba1 staining was identified within the cytoplasm of microglia (Figure 5E, inset); the positive cells were found diffusely distributed throughout the brain in infected animals (Figure 5D). In sham-inoculated animals, Iba1 Iba1-positive staining was present at lower percentages in comparison with the NiV-infected animals (Figure 6A). Most positive staining was found in the high dose groups of the IN (group 1 and 2, 10^5^ and 10^4^, respectively) and IP (group 4, 10^3^) routes (Figure 6A). Staining was evident in the most severe cases (Group 5, IP 10^2^) within the meninges and perivascular cuffs (Figures 5E,F).

There were no observed differences in the expression of GFAP positive staining between the different routes and doses of infection (Figure 6B), even in the most severe case (Group 5, IP 10^2^), and there were no abundant GFAP positive astrocytes associated with the lesions (Figures 5G,H). Staining of naïve animal brains showed lower positivity than that found in NiV-inoculated animals (Figure 5I).

**Figure 7:**
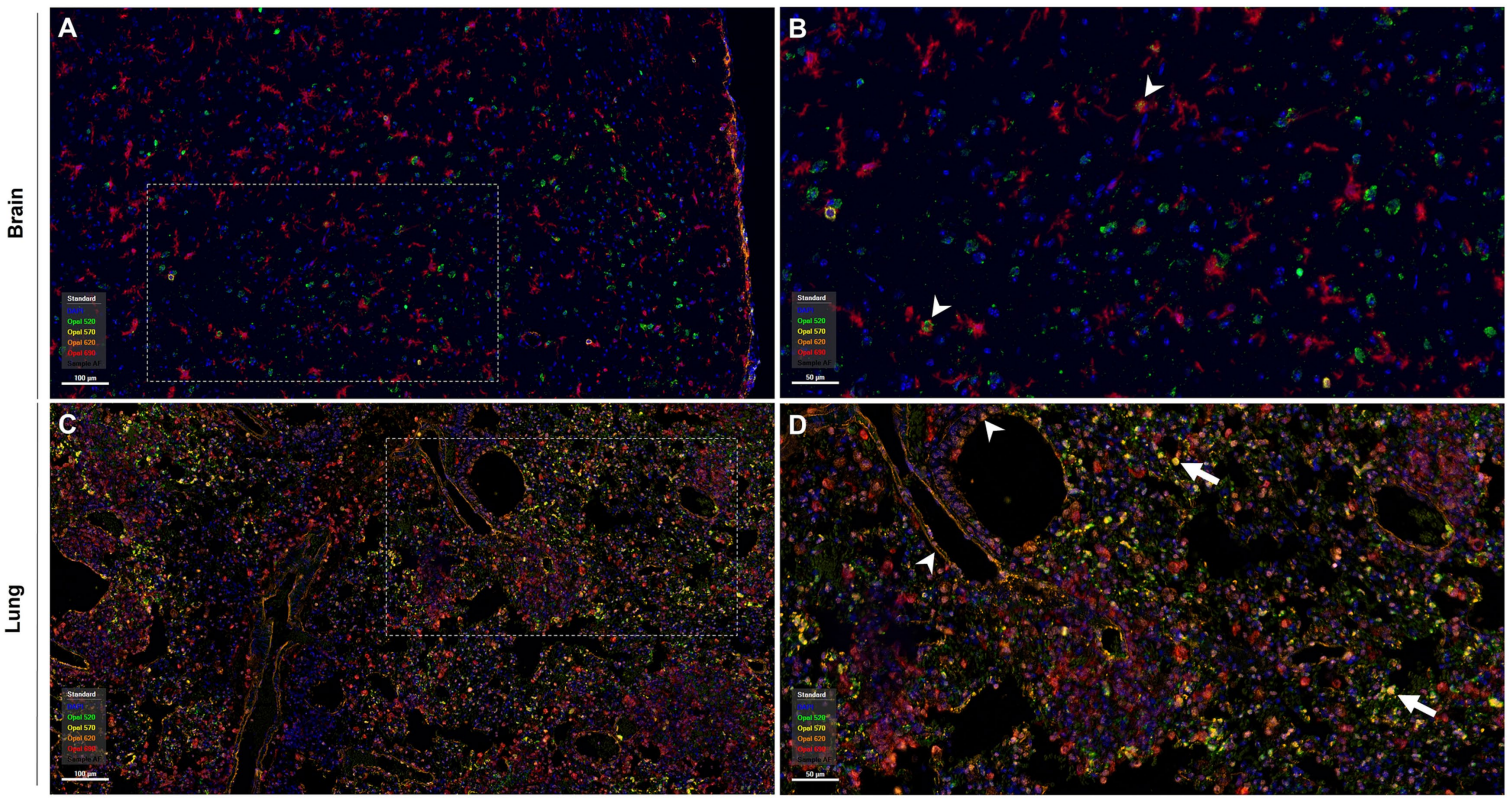
Representative images of multiplex immunofluorescence (mIF) in the lung and brain of a selected group 5 animal. **(A)** Section of the brain stained against blue, DAPI; green, NiV; yellow, CD3 (T lymphocytes); red, Iba1 (microglia); orange, GFAP (astrocytes). **(B)** Higher magnification of the mIF-stained brain. **(C)** Section of lung stained using mIF against blue, DAPI; green, NiV; yellow, CD3 (T lymphocytes); orange, Ephrin-B2; red, Iba1 (pneumocyte type II/macrophages). **(D)** Higher magnification of the mIF-stained lung. Scale bars **A,C** = 100 μm; **B,D** = 50 μm.

Table 4 shows quantification values of mIF in the brain. A high number of NiV^+^ cells were found within the midbrain (Figures 7A,B), which were associated with abundant Iba1, GFAP, and fewer CD3 positive cells (Table 4). Co-localisation shows NiV infection in macrophages (Iba1 positive) and fewer T lymphocytes (CD3 positive) (Table 4).

**Table 4:** Results of multiplex immunofluorescence (mIF) in the brain analysed using inForm and phenoptrReports.

NiV^+^/Iba1^+^	NiV^+^/CD3^+^	NiV^+^/GFAP^+^	NiV^+^	Iba1^+^	CD3^+^	GFAP^+^	Total
37	17	1	1,216	850	141	387	15,039

### 3.6 Cell phenotyping of lesions within the lung

**Figure 8:**
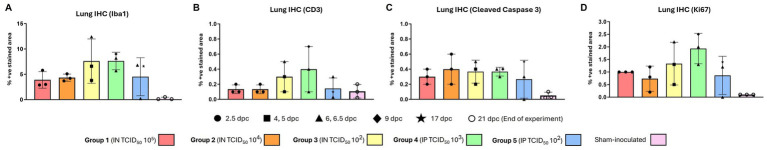
Quantitative results of Iba1, CD3, cleaved caspase-3, and Ki67 immunohistochemistry (IHC) in the lung of animals from different experimental groups. **(A)** Graph representing the quantification of Iba1 in the lung. **(B)** Graph representing the quantification of CD3 in the lung. **(C)** Graph representing the quantification of cleaved caspase 3 in the lung. **(D)** Graph representing the quantification of Ki67 in the lung. Column and whisker plots demonstrate the median and range with data points indicating individual animals. *N* = 3 animals per experimental group.

**Figure 9:**
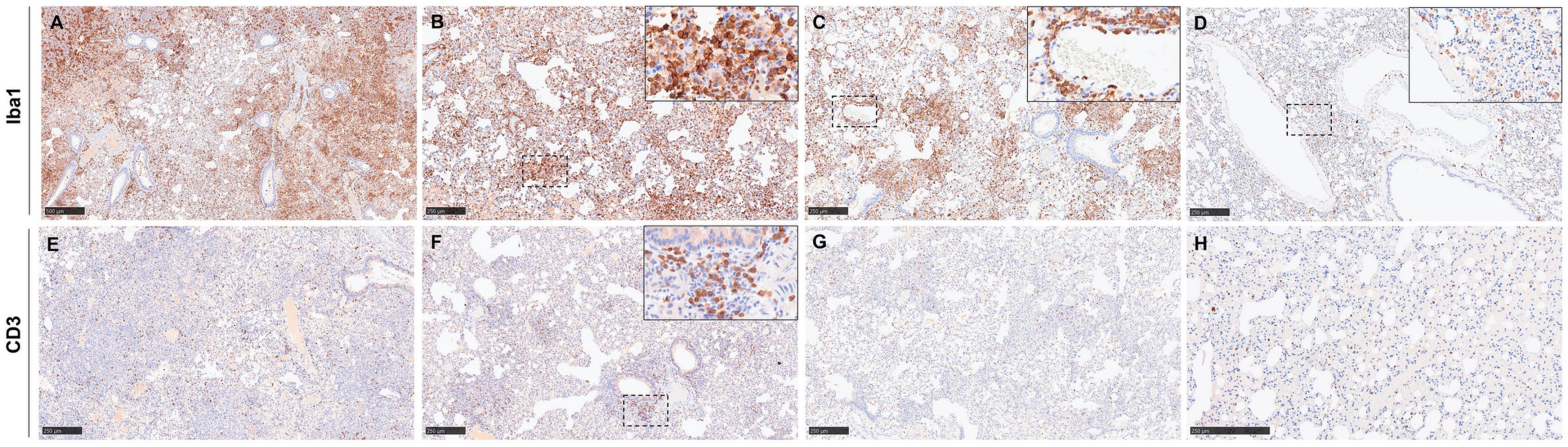
Representative images of immunohistochemistry (IHC) of the lung against Iba1 and CD3. **(A)** Lung from a group 3 animal stained against Iba1 showing intense positivity in areas of broncho-interstitial pneumonia. **(B)** Lung from a group 4 animal showing positive macrophages in areas of broncho-interstitial pneumonia. The inset shows higher magnification. **(C)** Lung from a group 5 animal demonstrating positive staining in areas of multifocal broncho-interstitial pneumonia and perivascular infiltration. The inset shows a higher magnification of perivascular infiltration. **(D)** Sham-inoculated sample with a few Iba1^+^ cells present. The inset shows higher magnification. **(E)** Lung from a group 3 animal showing scattered CD3^+^ cells. **(F)** Lung from a group 4 animal stained against CD3 showing positive cells in areas of multifocal broncho-interstitial pneumonia. The inset shows higher magnification. **(G)** Lung from a group 5 animal stained against CD3 showing fewer CD3^+^ cells. **(H)** Sham-inoculated sample with a few CD3^+^ cells present. Scale bars **B–H** = 250 μm; A = 500 μm.

The results of the image analysis of Iba and CD3 staining within the lung are shown in Figures 8A,B. Iba1-positive staining of macrophages was evident in areas of broncho-interstitial pneumonia (Figures 9A–C), as well as at the perivascular level (Figure 9C, inset). There was a higher percentage of Iba1-positive cells in samples from NiV-inoculated animals than in the negative controls (Figures 8A, 9D), especially in those from groups 3 (IN 10^2^) and 4 (IP 10^3^) (Figures 9A,B), which showed almost double of Iba1-positive area than the rest of the infected groups. The animals from the sham-inoculated group showed the presence of only a few macrophages (Figure 9D).

CD3-positive cells were found associated with broncho-interstitial pneumonia (Figures 9E–G) and also scattered throughout the pulmonary parenchyma (Figure 9H). Tissues from animals inoculated IN 10^2^ (group 3) and IP 10^3^ (group 4) demonstrated the highest percentage of CD3 staining (Figures 8B, 9E,F), similar to Iba1staining.

Image analysis of the mIF performed showed high levels of Ephrin B2 staining observed throughout the pulmonary parenchyma, particularly in endothelium at a perivascular level, in the alveolar walls and airways (Figures 7C,D, arrowheads and Table 5). Ephrin B2-positive cells showed high co-localisation with NiV antibody, indicating the presence of NiV in the cellular receptors of the lung cells (Table 5). Moreover, the receptor was observed in areas of severe broncho-interstitial pneumonia co-expressed with NiV and Iba1 positive cells (Table 5), indicating NiV infection in macrophages. Fewer CD3-positive T lymphocytes were observed co-located with NiV and Ephrin B2-positive cells (Figure 7B, arrows and Table 5).

**Table 5:** Results of multiplex immunofluorescence (mIF) in the lung analysed using inForm and phenoptrReports.

NiV^+^/EphB2^+^/Iba1^+^	NiV^+^/EphB2^+^/CD3^+^	NiV^+^/Iba1^+^	NiV^+^/CD3^+^	NiV^+^/EphB2^+^	NiV^+^	Iba1^+^	CD3^+^	EphB2^+^	Total
886	229	955	235	6,239	8,211	8,445	919	27,173	55,746

**Figure 10:**
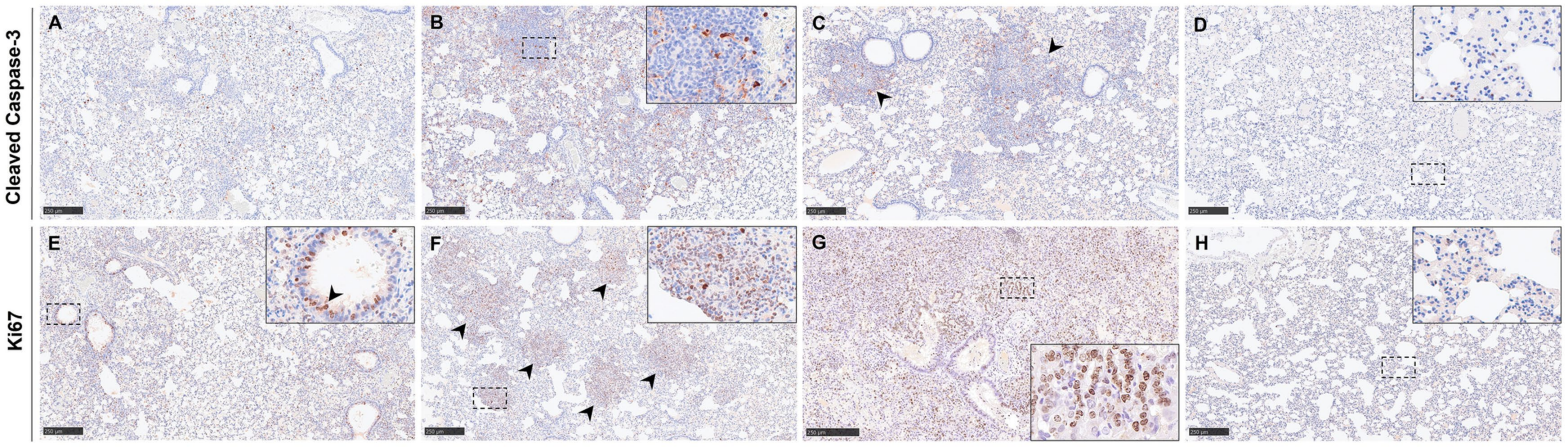
Representative images of immunohistochemistry (IHC) in the lung against cleaved caspase-3 (apoptosis) and Ki67 (proliferation). **(A)** Lung from a group 5 animal showing cleaved caspase-3^+^ cells diffusely distributed in the pulmonary parenchyma. **(B)** Lung from a group 2 animal showing apoptotic cells in areas of broncho-interstitial pneumonia. Inset shows a higher magnification of apoptotic macrophage-like and lymphocyte-like cells. **(C)** Lung from a group 3 animal showing apoptotic cells in areas of broncho-interstitial pneumonia (arrowheads). **(D)** Lung from a sham-inoculated group animal showing a few positive cells stained against cleaved caspase-3. The inset shows higher magnification. **(E)** Lung from a group 2 animal showing Ki67^+^ cells diffusely distributed in the pulmonary parenchyma and in the bronchiolar epithelium. The inset shows a higher magnification of positive epithelial cells. **(F)** Lung from a group 4 animal showing proliferating Ki67^+^ cells in regions of broncho-interstitial pneumonia (arrowheads). The inset shows higher magnification. **(G)** Lung section from a group 3 animal showing intense staining against Ki67 in an area of interstitial pneumonia. The inset shows a higher magnification of pneumocytes type II. **(H)** Lung section from a sham-inoculated group animal showing a few positive cells stained against Ki67. The inset shows higher magnification. Scale bars **(A–H)** = 250 μm.

The quantification of apoptotic cells in the lung showed little to no difference in the expression of cleaved caspase-3 between the different routes of inoculation and dose (Figure 8C). In the sham-inoculated group, there were considerably fewer cleaved caspase-3 positive cells in comparison with the infected animals (Figures 8C, 10D). Cleaved caspase-3 expression was diffusely distributed throughout the pulmonary parenchyma (Figures 10A–C), especially in areas of broncho-interstitial pneumonia (Figures 10B,C, arrowheads). Cleaved caspase-3 positive was mainly detected in mononuclear cells (Figure 10B, inset).

The Ki67 staining (marker of cell proliferation) was observed within areas of broncho-interstitial pneumonia (Figure 10F, inset) and airways (Figure 10E, inset and arrowheads). Ki67 positive cells were mainly inflammatory cells (mononuclear and polymorphonuclear cells) (Figure 10F, inset), pneumocytes type II (Figure 10G, inset), and epithelial cells from the airways (Figure 10E, inset). Of note, there were more Ki67 positive cells in lung samples from animals inoculated IN 10^2^ (group 3) and IP 10^3^ (group 4) (Figures 8D, 10F,G), like the expression of Iba1 and CD3. Samples from the sham-inoculated group stained against Ki67 showed positivity only in a few cells throughout the pulmonary parenchyma (Figures 8D, 10H).

**Figure 11:**
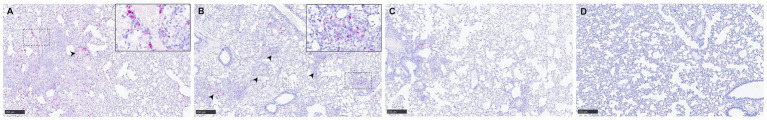
Representative images of IL-6 RNAscope in the lung. **(A)** Lung from a group 3 animal demonstrating IL-6 staining in an area of pneumonia and at a perivascular level (inset and arrowhead). **(B)** Lung from a group 5 animal showing IL-6 mRNA in areas of broncho-interstitial pneumonia (arrowheads). The inset shows a higher magnification. **(C)** Lung from a group 2 animal showing low levels of IL-6 mRNA. **(D)** Lung from a sham-inoculated group animal showing no IL-6 mRNA.

IL-6 mRNA was especially expressed in lung samples from animals inoculated IN 10^2^ (group 3) and IP 10^3^ (group 4) (Supplementary Figure 3; Figure 11A). Only occasional cells were positive for IL-6 mRNA detection in samples from animals inoculated IN with a high-dose group (10^4^ and 10^5^) (Figure 11C) and a sham-inoculated group (Figure 11D). IL-6-positive cells were found in areas of broncho-interstitial pneumonia (Figures 11A,B, arrowheads) and at the perivascular level (Figure 11A, arrowhead and inset).

## 4 Discussion

NiV can be found on the WHO R&D Blueprint list as a major public health concern with an urgent need for attention and expedited research and countermeasure development (5, 6). Not only can NiV be transmitted from animals and contaminated foods to humans, but it can also be transmitted from human to human (18, 48, 49). With a case fatality rate ranging from 40–75% (6), due to its high pathogenicity and ability to cause significant economic losses to local communities, NiV remains a potential threat to humans. With a lack of licensed vaccines and countermeasures, as well as a natural reservoir (*Pteropodidae*) that is widely geographically distributed, there is a significant risk of future spillover events that increases the urgency of further NiV research (8–10, 50).

There are two major NiV strains used for preclinical studies: NiV-M and NiV-B (15, 16). NiV-B has been associated with recent NiV outbreaks in the Dhaka district of Bangladesh and Kerala state in India (42, 51). Kerala has experienced its ninth outbreak in 7 years (51–53), with two occurring in 2024 alone and four confirmed cases in July 2025 (51). Moreover, this NiV strain is suggested to be more pathogenic in humans than NiV-M (CFR NiV-B: 70.97%, CFR NiV-M: ~40%) (33, 37, 42, 43). Due to the recent appearance of new NiV-B cases and following the UK national organisation (NC3r’s) 3Rs recommendations to reduce, refine, and replace animal experimentation, we utilised archived golden Syrian hamster samples from previous animal experiments to delve into the understanding of NiV-B pathogenesis and compare with our previously published NiV-M results (27, 36).

DeBuysscher et al. (35) first examined NiV-B infection in an animal model in 2013. Golden Syrian hamsters infected with NiV-B showed a delay in disease progression and increased survival rate compared with NiV-M infected animals, independent of the administration route (35). Similar results were observed in a recent study where NiV-B administered IN was 60% lethal at the highest dose evaluated (TCID_50_ 10^6^), whereas 100% lethality was achieved with the same dose of NiV-M (54). In a recent publication from Davies et al. (32), where they analysed 19 independent studies using NiV-M or NiV-B hamsters, inoculated IN and IP were compared, and a higher lethality of NiV-B than NiV-M following IN inoculation (TCID_50_ 10^6^) was observed. This study, along with our previous NiV-M (27) results, shows similar results. As early as 2 days post-challenge, all animals infected with NiV-B met humane endpoints, whereas those infected with NiV-M met humane endpoints approximately 7 dpc (TCID_50_ 10^5^ and 10^4^) (Supplementary Figure 4). Davies et al. (32) reported the opposite trend when NiV is administered via IP, suggesting strain- and route-dependent effects.

Our previous results showed that animals infected with NiV-M exhibited both respiratory and neurological signs (ataxia and paralysis, among others), especially those infected via IP (27). In our study, except for one animal that survived until nine dpc (group 4, IP TCID_50_ 10^3^), none of the hamsters experienced neurological signs, with respiratory disease (dyspnoea/tachypnoea) being the only clinical presentation before meeting the humane endpoint. This finding could suggest that animals would have had a fatal outcome due to the respiratory disease before developing other clinical manifestations. Although this fact, together with the higher fatality, would be in accordance with what is observed during NiV-B human infection, it has not been observed in other studies with hamsters (35, 54). In fact, in the meta-analysis performed by Davies et al. (32), the neurological disease was particularly observed in IP inoculated NiV-B hamsters, where the respiratory signs were rarely seen.

The golden Syrian hamster NiV-M model was seen to have similar neuro and respiratory pathology as that described in humans (36, 37, 55). In our study with NiV-B, although some mild histopathological lesions were observed in the rest of the organs, the main lesion was present in the lung. Only the animal that survived until day 17 of the study presented severe lesions in the brain, suggesting that animals die due to the respiratory disease before developing neurological lesions. This could be explained by the virus’ infection initially targeting the respiratory system and later developing into the neurological disease following the recovery from the respiratory phase (56). Similar lesions were observed in the lung from NiV-B-infected hamsters compared with ones infected with NiV-M, previously published (36), independent of the dose and route of infection. In both cases, lesions consisted of moderate to severe multifocal to coalescing areas of broncho-interstitial pneumonia, characterized by thickening of the alveolar walls and type II pneumocyte hyperplasia with necrosis of alveoli and bronchiolar epithelium, accompanied by alveolar macrophages (Iba1 positive), T cell infiltration (CD3 positive), and few heterophils. Baseler et al. (33) reported more severe rhinitis and broncho-interstitial pneumonia 2 days after infection with NiV-B compared with NiV-M in the hamster model, but these could not be detected by day four after inoculation (33). On the contrary, DeBuysscher et al. (35) observed that pulmonary histopathological changes appeared earlier in high-dose infected NiV-M hamsters compared to NiV-B animals.

These observed differences could be due to the different experimental settings applied in each site, which makes it more difficult to compare the studies. Moreover, the sex and age of the animals could have influenced the clinical and pathological outcomes of this present study compared to other published studies. Although previous studies demonstrated no significant sex-based differences in the clinical outcomes and humoral response in the NiV Syrian hamster model (32, 57), it is something that we should take into account for future studies. In the meta-analysis performed by Davies et al. (32), a higher lethality in animals <9 weeks was observed in comparison with those ≥9 weeks. In our study, animals were >8 weeks when they were infected. Considering the observed lethality, the age of the animals could have influenced the clinical and pathological outcomes.

In our study, pulmonary lesions were associated with a high amount of viral RNA detected by *in situ* hybridization (RNAscope technique), with a similar distribution trend in comparable groups of NiV-M inoculated animals (36). We selected this technique over immunohistochemistry due to its superior sensitivity and specificity while preserving tissue morphology (58). Only mild histological lesions with no viral RNA detected by RNAscope technique were visible in the brain, liver, and spleen from IN-inoculated animals (TCID_50_ 10^5^ and 10^4^, humane endpoint 2–4 dpc). This fact suggests that although viral biodistribution takes place earlier after the infection (32, 35), the amount of NiV RNA is under the limit of detection of this technique. In IP-infected animals, greater quantities of NiV were found in these tissues due to the viruses ability to directly systemically circulate as opposed to having to penetrate the aerodigestive tract barrier (31).

In order to assess cellular death, the cleaved caspase-3 immunolabeling method was used, demonstrating the activation of the caspase-dependent apoptotic pathway in the NiV-infected lung. Cleaved caspase-3 was detected in the nuclei and cytoplasm of macrophages, lymphocytes, neutrophils, and apoptotic bodies from areas of broncho-interstitial pneumonia (Iba1 and CD3 positive). Due to the lack of lesions and the presence of non-specific staining in the brain, this marker was not evaluated in this organ. There is a lack of research in this regard, so it would be interesting for future studies to analyse the mechanism of cellular death that takes place in target organs during NiV infection. Moreover, we evaluated the expression of the marker Ki67 in order to study the cell proliferation in the injured pulmonary areas. Unsurprisingly, Ki67, Iba1, and CD3 markers showed the same pattern of expression among the different infection groups, demonstrating the proliferation of immune cells involved in broncho-interstitial pneumonia (macrophages, T lymphocytes, and type II pneumocytes) and the regeneration of the epithelia and lung parenchyma.

Regarding the expression of the proinflammatory cytokine IL-6 detected by RNAscope, we previously observed an upregulation of this cytokine in the lung from animals inoculated by the IN route at high doses (36). In this study, minimal IL-6 mRNA was observed in the pulmonary parenchyma of these comparable groups, suggesting a different pattern of activation of the local immune response between the two strains, as was demonstrated by DeBuysscher et al. (35) via qRT-PCR. In NiV-M-infected animals, IL-6 mRNA was associated with inflammatory cells within areas of broncho-interstitial pneumonia and vasculitis (36). IL-6 is a key activator of CD4 and CD8 T cells, and the recruitment of these cells could contribute to the striking vasculitis observed in these NiV-M-infected animals (35, 36, 59–61). In this sense, NiV-M could be infecting different cell types more effectively (such as endothelial cells and immune cells) that may produce a distinct cytokine and chemokine environment. A detailed analysis of the NiV-M and NiV-B immune responses would be highly valuable for obtaining a comprehensive picture of the immune response to NiV infection in the hamster model.

It is widely described that Ephrin B2 acts as the main entry receptor for NiV and that it is expressed in endothelial cells and neurons (33, 39, 41, 62); however, to the authors’ knowledge, there are no images available of its expression during NiV infection *in vivo*. Herein, we demonstrate its expression in endothelial cells, neurons, smooth muscle, and epithelial cells, which is consistent with the cellular tropism for NiV. Although image analysis of the staining was not performed due to the ubiquitous distribution of the marker, no striking differences were found between the expression and distribution pattern in tissues from healthy and infected hamsters. Moreover, we developed an algorithm to analyse mIF, which allowed us to identify NiV-infected macrophages/type II pneumocytes and T cells expressing Ephrin B2.

In conclusion, due to the recent NiV outbreaks associated with NiV-B and its higher virulence in humans, we aimed to characterize the clinical response and histopathological lesions present in the NiV-B golden Syrian hamster model and compare with previous results published with the NiV-M strain. In our study, an absence of neurological signs and lesions was observed in NiV-B compared with NiV-M infected animals, suggesting that animals may die due to the respiratory disease before developing other clinical manifestations and lesions. We applied an array of histopathological techniques to further characterize the lesions, cell death, cytokine response, and NiV receptor expression in the lung of NiV-B golden Syrian hamster model.

## Data Availability

The raw data supporting the conclusions of this article will be made available by the authors, without undue reservation.
