# Supplementary material for: Histopathological and immunohistochemical characterization of lesions in the golden Syrian hamster model of Nipah virus infection (Bangladesh strain)
Source: Front Vet Sci. 2026 Jan 19;12:1708412. doi: 10.3389/fvets.2025.1708412 (PMC12862940; doi:10.3389/fvets.2025.1708412)
Supplement: Supplementary file 1 [file Supplementary_file_1.docx]

**Supplementary Figures**

**
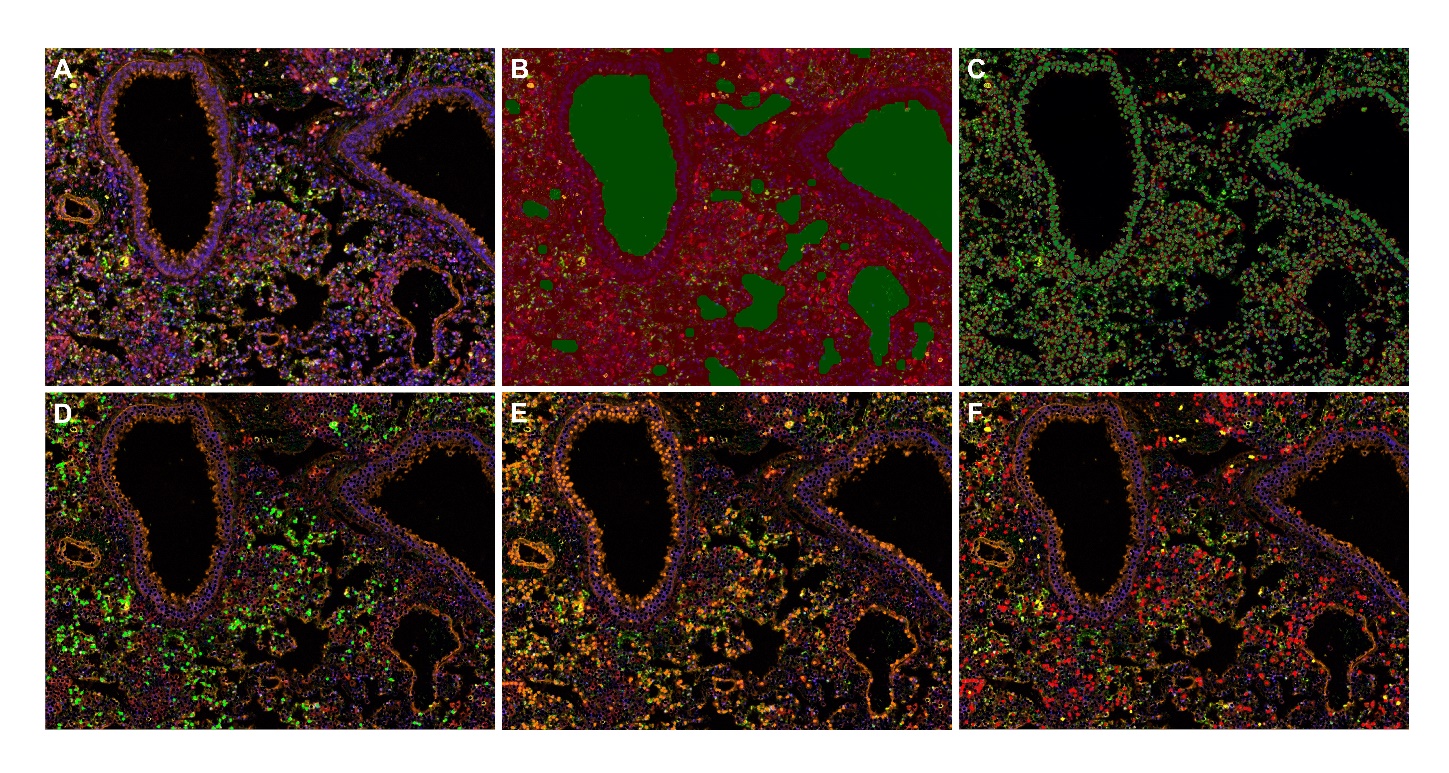
Supplementary Figure 1. Methodology applied to perform the image analysis in the lung with the inForm software.** **(A)** Multiplex immunofluorescence in selected field (blue, dapi; green, NiV; orange, EphB2 (NiV receptor); yellow, CD3 (T lymphocytes); red, Iba1 (pneumocyte type II/ macrophages). **(B)** Tissue segmentation performed with the inForm Software (‘tissue’ red; ‘non tissue’ green). **(C)** Cell segmentation performed with the inForm Software. **(D)** Cell phenotyping performed with the inForm Software of Nipah schema (green, NiV; black, other). **(E)** Cell phenotyping performed with the inForm Software of Receptor schema (orange, EphB2; black, other). **(F)** Cell phenotyping performed with the inForm Software of Marker schema (yellow, CD3; red, Iba1; black, other).

**
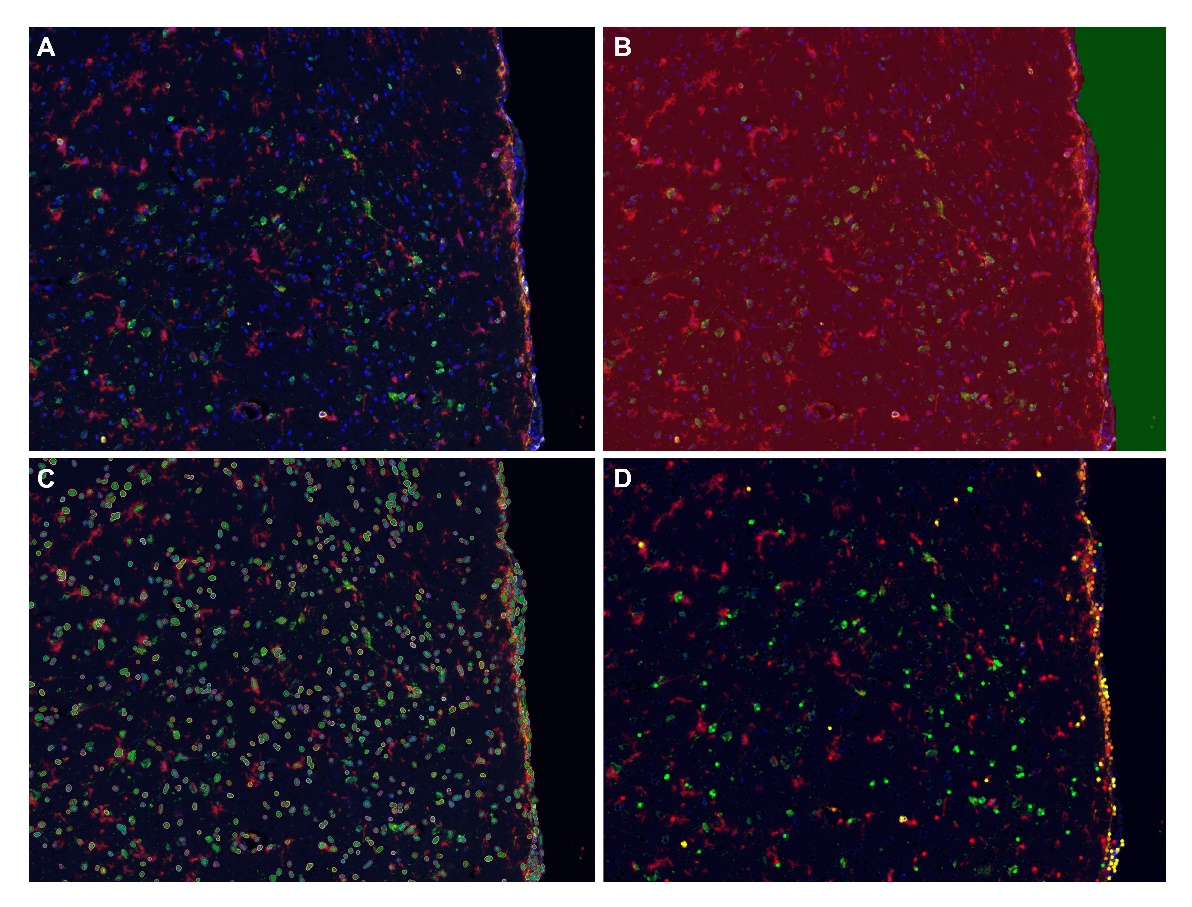
**

**Supplementary Figure 2. Methodology applied to perform the image analysis in the brain with the inForm Software.** **(A)** Multiplex immunofluorescence in selected field (blue, dapi; green, NiV; yellow, CD3 (T lymphocytes); red, Iba1 (pneumocyte type II/ macrophages); orange, GFAP (astrocytes)). **(B)** Tissue segmentation performed with the inForm Software (‘tissue’ red; ‘non tissue’ green). **(C)** Cell segmentation performed with the inForm Software. **(D)** Cell phenotyping performed with the inForm Software.


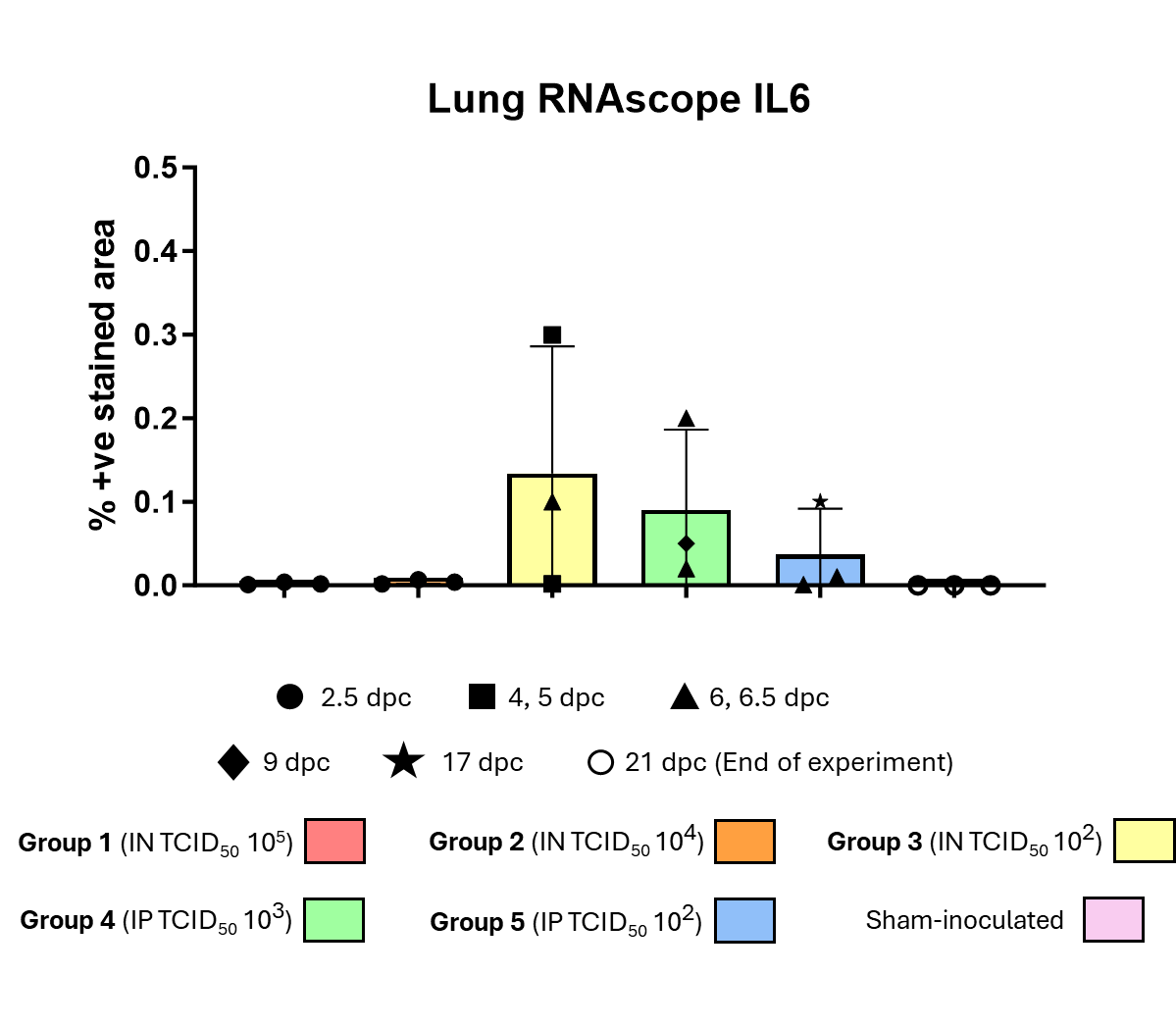


**Supplementary Figure 3. Quantitative results of RNAscope against IL-6 in the lung of animals from different experimental groups following image analysis.** Column and whisker plots demonstrate the median and range with data points indicating individual animals. *N=3* animals per experimental group.

**Supplementary Figure 4. Kaplan–Meier survival plot comparing NiV-B and NiV-M intranasally (IN) infected hamsters with different doses** (TCID_50_ 10^5^ and TCID_50_ 10^4^). Solid lines show NiV-B data and dotted lines show NiV-M data. NiV-M *N*=3 animals per experimental group, NiV-B TCID_50_ 10^5^ *N*=10 animals per experimental group and TCID_50_ 10^4^ *N*=4 animals per experimental group.
